# Supplementary material for: Predicting individual long-term prognosis of spatial neglect based on acute stroke patient data
Source: Brain Commun. 2025 Jan 31;7(1):fcaf047. doi: 10.1093/braincomms/fcaf047 (PMC11814933; doi:10.1093/braincomms/fcaf047)
Supplement: fcaf047_Supplementary_Data [file fcaf047_supplementary_data.pdf]

Supplementary Material

Predicting individual long-term prognosis of spatial neglect based on acute stroke patient data

Lisa Röhrig,[1] Daniel Wiesen,[1] Dongyun Li,[1†] Christopher Rorden,[2] Hans-Otto Karnath[1,2]

Affiliations:

[1] Center of Neurology, Division of Neuropsychology, Hertie Institute for Clinical Brain Research, University of Tübingen, Tübingen, Germany

[2] Department of Psychology, University of South Carolina, Columbia, SC, USA

[†] Now at: Department of Child Health Care, Children's Hospital of Fudan University, Shanghai, China

Supplementary Methods and Results

Target variables

Calculation of recovery:

We calculated the effectiveness of recovery based on a previously reported formula (adapted from Grasso et al., 2005; Shah et al., 1990), in which we used the value zero for the "maximum score" since a CoC or copying error of zero represents no deficit. Note that a recovery of greater than 100% was set to 100% before averaging. The formula was as follows:

$$recovery = \left(\frac{chronic\ score - acute\ score}{0 - acute\ score}\right) * 100\%$$

Controls' recovery score:

For the recovery score, it is not evident whether control patients behave like patients who fully recovered or who did not recover at all. Therefore, we tested in a pilot investigation whether setting the control patients to 0% or to 100% recovery would result in smaller prediction errors. When the algorithm was trained with controls assigned to 100% recovery, we found more accurate predictions for neglect patients (see main manuscript). Nevertheless, we report results from prediction models that were trained with control's recovery score being set to 0% in the following:

The variable *acute behavior* resulted in the lowest prediction error among all single predictors (MSE = 0.083 ± SD 0.156, R^2 = -0.51, r = -0.13 with p = 0.41; Supplementary Fig. 1A). As for the z-score difference, the feature selection for the recovery score detected a combination of variables that outperformed the most predictive single predictor. In addition to the *acute behavior*, feature selection selected the variable *WB-%-PCs*, i.e. the lesion location described by PCs cumulatively explaining > 98% of the imaging variance derived from the whole-brain lesion maps (MSE = 0.081 ± SD 0.090, R^2 = -0.46, r = 0.02 with p = 0.91; Supplementary Fig. 1B). The full model with the lesion location variable *ROI-%-PCs* served as the best performing full model (MSE = 0.111 ± SD 0.110, R^2 = -1.02, r = -0.04 with p = 0.79; Supplementary Fig. 1A).

Although the model including the predictor *acute behavior* and the lesion location variable *WB-%-PCs* yielded the lowest error for predicting effectiveness of recovery (when control patients were set to 0% recovery), the overall performance was not sufficient to be meaningful. This is illustrated by a very poor model fit for chronic patients (r = 0.03, p = 0.92), although recovered patients were moderately well predicted (r = 0.48, p = 0.007; Supplementary Fig. 1C). Findings revealed that none of the models that

aimed to predict the effectiveness of recovery was able to explain some proportion of total variance (no positive-valued R^2). To summarize, none of the models predicting neglect recovery was detected to achieve meaningful predictions, which also weakens the potential prognostic value of the included predictors.

Predictor variables

Selection of principal components:

For each target variable and map variant separately, we tried different thresholds of cumulative explained variance and used the winning threshold (i.e., that produced the most accurate predictions) for further analyses (*chronic z-score*: WB lesion map 98% [N = 50], ROI overlap 90% [N = 19]; *difference*: WB lesion map 98% [N = 50], ROI overlap 100% [N = 71]; *recovery*: WB lesion map 100% [N = 71], ROI overlap 100% [N = 71]).

Again, for each target variable and map variant separately, we tried three different feature selection filter methods (minimum redundancy maximum relevance algorithm [MRMR], univariate feature ranking for regression using F-tests [F-test], neighborhood component analysis [NCA]). We then selected the five most important components that were most strongly associated with the target variable, identified by the winning filter method (*chronic z-score*: WB lesion map – F-test [PCs 13, 5, 36, 29, 55], ROI overlap – NCA [PCs 14, 13, 1, 2, 38]; *difference*: WB lesion map – NCA [PCs 3, 7, 26, 10, 11], ROI overlap – F-test [PCs 14, 58, 48, 2, 13]; *recovery*: WB lesion map – NCA [PCs 3, 1, 2, 6, 55], ROI overlap – F-test [PCs 50, 62, 25, 18, 4]).

Supplementary Figures

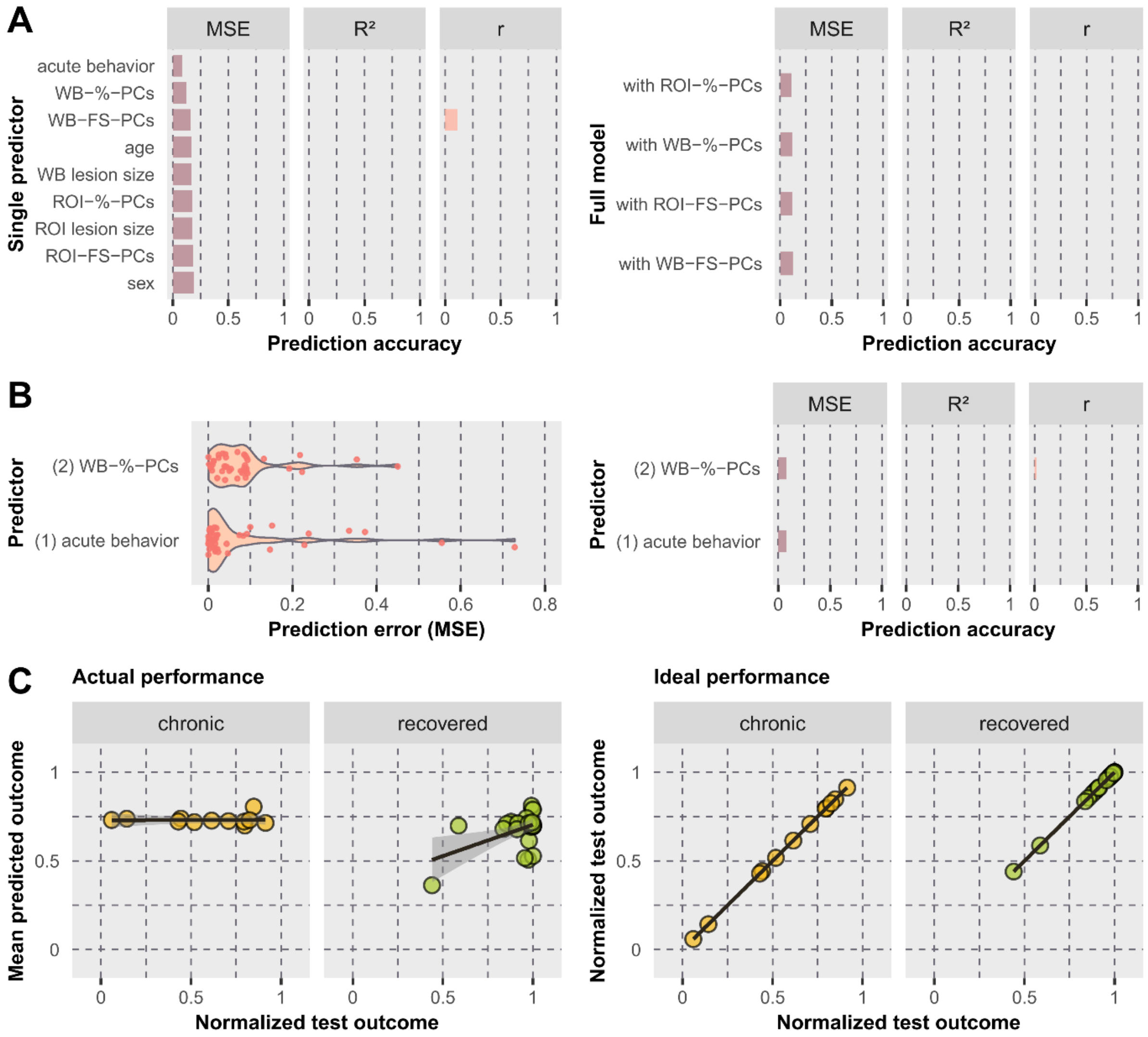

Supplementary Figure 1. Model performances when predicting the effectiveness of recovery. Prediction was performed using Support Vector Regression (SVR) with repeated nested cross-validation. Recovery scores of control patients were set to 0% (compare Fig. 6 within the main manuscript for results when controls' recovery was set to 100%). **(A)** Results are illustrated for single predictors (left) and full models (right), and **(B)** for predictors selected by feature selection. **(C)** Test scores versus predicted scores are shown for the best performing model (acute behavior, WB-%-PCs). For all further details, see legend of Figure 3 within the main manuscript.

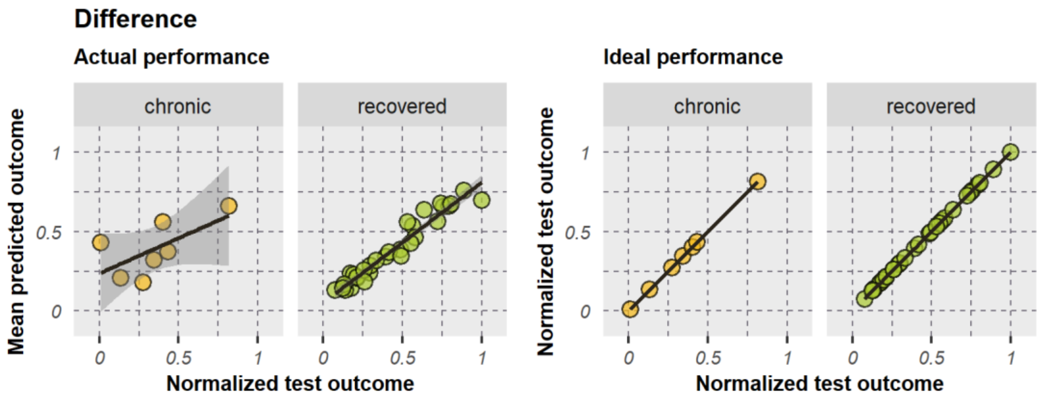

Supplementary Figure 2. Model fit of the best predictive model (when predicting the z-score difference). Prediction was performed using Support Vector Regression (SVR) with repeated nested cross-validation. Model performance is illustrated for chronic (*N* = 7) and recovered patients (*N* = 30); chronic neglect patients (*N* = 5), who were also included in the creation of the chronic neglect ROI (Karnath et al., 2011), were excluded (compare with Fig. 5C in the main manuscript).

Supplementary Tables

Supplementary Table 1. Cross-validated prediction performance for models using different chronic z-scores for control patients.

Target variable to predict	Control's chronic z-score = 0	Control's chronic z-score = acute z-score	Absolute delta
Chronic z-score	$R^2 = 0.548$	$R^2 = 0.543$	$\Delta R^2 = 0.005$ (0.5%)
Difference	$R^2 = 0.662$	$R^2 = 0.650$	$\Delta R^2 = 0.012$ (1.2%)

Note. For the chronic z-scores of control patients, we used the averaged acute z-score of controls (i.e. mean acute z-score = 0; see main manuscript and Supplementary Tables 2-3 below). We repeated the most accurate models with controls' chronic z-score being set to the individual acute z-scores. Models performed almost stable, with an absolute delta of about 1% explained variance.

Supplementary Table 2. Model performances for predicting the chronic z-score.

Model	Predictor(s)	MSE	R²	r	*p*(r)
Single/FS-1	Acute behavior	0.0543	-0.101	-0.139	n.s.
	**Age**	**0.0490**	**0.007**	**0.115**	**n.s.**
	Sex	0.0501	-0.015	-0.539	***
	WB lesion size	0.0505	-0.025	0.036	n.s.
	**ROI lesion size**	**0.0372**	**0.246**	**0.525**	*****
	WB-%-PCs	0.0517	-0.048	-0.542	***
	**WB-FS-PCs**	**0.0223**	**0.548**	**0.771**	*****
	ROI-%-PCs	0.0549	-0.114	-0.021	n.s.
	**ROI-FS-PCs**	**0.0460**	**0.068**	**0.271**	**n.s.**
FS-2	Acute behavior	0.0418	0.153	0.410	**
	Age	0.0305	0.381	0.642	***
	Sex	0.0400	0.189	0.473	**
	WB lesion size	0.0470	0.047	0.274	n.s.
	ROI lesion size	0.0360	0.271	0.563	***
Full model	WB-%-PCs	0.0517	-0.048	-0.153	n.s.
	**WB-FS-PCs**	**0.0465**	**0.058**	**0.263**	**n.s.**
	ROI-%-PCs	0.0514	-0.041	-0.063	n.s.
	ROI-FS-PCs	0.0554	-0.124	-0.069	n.s.

Note. Prediction accuracies are presented for models predicting the chronic z-score (chronic neglect severity). Cross-validated performance was measured using the mean squared error (MSE), coefficient of determination (R^2), and Pearson correlation coefficient (r, with its corresponding significance value *p*; n.s. – not significant, * – $p < 0.05$, ** – $p < 0.01$, *** – $p < 0.001$). Models used either single predictors ("single"), combinations of thereof by applying forward sequential feature selection ("FS-1" represents the first iteration, "FS-2" represents the second iteration testing the winning predictor of the first iteration and a second variable), and full models using all variables but only one lesion location variable. Results of predictive variables (that explain some variance or improve the model) are in bold type, whereas the row of the selected predictor (i.e., winning model) is highlighted in grey. Note that an ideal model would achieve values of $MSE = 0$, $R^2 = 1$, $r = 1$ (a negative R^2 and a negative r represents a model with a very poor model fit).
Abbreviations: WB – whole-brain, ROI – (chronic neglect) region of interest, % – PCs selected according to a certain proportion of cumulatively explained variance, FS – PCs identified by a filter feature selection approach (five most important PCs selected), PCs – principal components.

Supplementary Table 3. Model performances for predicting the z-score difference.

Model	Predictor(s)	MSE	R^2	r	*p*(r)
Single/FS-1	**Acute behavior**	**0.0263**	**0.588**	**0.768**	*******
	Age	0.1170	-0.830	-0.085	n.s.
	Sex	0.1303	-1.037	0.007	n.s.
	WB lesion size	0.0853	-0.335	0.231	n.s.
	ROI lesion size	0.1522	-1.380	-0.015	n.s.
	WB-%-PCs	0.0693	-0.084	0.310	*
	WB-FS-PCs	0.0797	-0.247	0.294	n.s.
	ROI-%-PCs	0.0772	-0.207	0.161	n.s.
	ROI-FS-PCs	0.0960	-0.501	0.230	n.s.
FS-2	**Age**	**0.0251**	**0.608**	**0.780**	*******
	Sex	0.0283	0.557	0.747	***
	WB lesion size	0.0267	0.582	0.763	***
	**ROI lesion size**	**0.0239**	**0.626**	**0.792**	*******
	WB-%-PCs	0.0278	0.566	0.753	***
	**WB-FS-PCs**	**0.0262**	**0.590**	**0.769**	*******
	**ROI-%-PCs**	**0.0251**	**0.607**	**0.784**	*******
	**ROI-FS-PCs**	**0.0223**	**0.651**	**0.807**	*******
FS-3	Age	0.0257	0.598	0.776	***
	Sex	0.0254	0.602	0.777	***
	**WB lesion size**	**0.0221**	**0.655**	**0.811**	*******
	**ROI lesion size**	**0.0216**	**0.662**	**0.814**	*******
FS-4	Age	0.0235	0.632	0.797	***
	Sex	0.0250	0.609	0.781	***
	WB lesion size	0.0254	0.603	0.777	***
Full model	**WB-%-PCs**	**0.0301**	**0.530**	**0.728**	*******
	**WB-FS-PCs**	**0.0299**	**0.533**	**0.731**	*******
	**ROI-%-PCs**	**0.0289**	**0.548**	**0.741**	*******
	**ROI-FS-PCs**	**0.0280**	**0.563**	**0.752**	*******

Note. Prediction accuracies are presented for models predicting the chronic z-score (chronic neglect severity). Cross-validated performance was measured using the mean squared error (MSE), coefficient of determination (R^2), and Pearson correlation coefficient (r, with its corresponding significance value *p*; n.s. – not significant, * – $p < 0.05$, ** – $p < 0.01$, *** – $p < 0.001$). Models used either single predictors ("single"), combinations of thereof by applying forward sequential feature selection ("FS-1" represents the first iteration, "FS-2" represents the second iteration testing the winning predictor of FS-1 and a second variable, "FS-3" represents the third iteration testing the winning combination of FS-2 and a third variable, and so on), and full models using all variables but only one lesion location variable. Results of predictive variables (that explain some variance or improve the model) are in bold type, whereas rows of the selected predictors (i.e., winning models) are highlighted in grey. Note that an ideal model would achieve values of $MSE = 0$, $R^2 = 1$, $r = 1$ (a negative R^2 and a negative r represents a model with a very poor model fit).
Abbreviations: WB – whole-brain, ROI – (chronic neglect) region of interest, % – PCs selected according to a certain proportion of cumulatively explained variance, FS – PCs identified by a filter feature selection approach (five most important PCs selected), PCs – principal components.

Supplementary Table 4. Model performances for predicting the effectiveness of recovery.

Model	Predictor(s)	MSE	R^2	r	*p*(r)
Single/FS-1	Acute behavior	0.0698	-0.120	-0.354	*
	Age	0.0676	-0.084	0.223	n.s.
	Sex	0.0680	-0.090	-0.046	n.s.
	WB lesion size	0.0662	-0.062	0.096	n.s.
	ROI lesion size	0.0857	-0.374	0.265	n.s.
	WB-%-PCs	0.0673	-0.079	-0.393	*
	WB-FS-PCs	0.0685	-0.098	-0.144	n.s.
	ROI-%-PCs	0.0687	-0.102	-0.169	n.s.
	ROI-FS-PCs	0.0665	-0.067	0.124	n.s.
FS-2	Acute behavior	0.0701	-0.123	-0.310	*
	Age	0.0687	-0.101	-0.095	n.s.
	Sex	0.0699	-0.121	-0.136	n.s.
	ROI lesion size	0.0704	-0.129	0.002	n.s.
	WB-%-PCs	0.0685	-0.098	-0.395	**
	WB-FS-PCs	0.0695	-0.115	-0.182	n.s.
	ROI-%-PCs	0.0699	-0.120	-0.274	n.s.
	ROI-FS-PCs	0.0688	-0.104	0.020	n.s.
Full model	WB-%-PCs	0.0710	-0.139	-0.431	**
	WB-FS-PCs	0.0732	-0.174	-0.452	**
	ROI-%-PCs	0.0684	-0.096	-0.311	*
	ROI-FS-PCs	0.0706	-0.132	-0.202	n.s.

Note. Prediction accuracies are presented for models predicting the chronic z-score (chronic neglect severity). Cross-validated performance was measured using the mean squared error (MSE), coefficient of determination (R^2), and Pearson correlation coefficient (r, with its corresponding significance value *p*; n.s. – not significant, * – $p < 0.05$, ** – $p < 0.01$, *** – $p < 0.001$). Models used either single predictors ("single"), combinations of thereof by applying forward sequential feature selection ("FS-1" represents the first iteration, "FS-2" represents the second iteration testing the winning predictor of the first iteration and a second variable), and full models using all variables but only one lesion location variable. Note that none of the models could explain some variance. The row of the selected predictor (i.e., winning model) is highlighted in grey. Note that an ideal model would achieve values of $MSE = 0$, $R^2 = 1$, $r = 1$ (a negative R^2 and a negative r represents a model with a very poor model fit). Control patients (without spatial neglect) were assigned recovery scores of 100%.
Abbreviations: WB – whole-brain, ROI – (chronic neglect) region of interest, % – PCs selected according to a certain proportion of cumulatively explained variance, FS – PCs identified by a filter feature selection approach (five most important PCs selected), PCs – principal components.

Supplementary Table 5. Brain regions and tracts included in principal components predictive for neglect prognosis.

Atlas	Components of whole-brain lesion maps			Components of ROI-based overlaps		
	**Gyrus/Tract**	**Label**	***N***	**Gyrus/Tract**	**Label**	***N***
Gray matter (BNA)	MTG	88	4248	MTG	88	2524
	Putamen	230	2061	Putamen	230	2106
	Insular Gyrus	164	1564	STG	80	1023
	Insular Gyrus	172	1488	Globus Pallidus	222	1013
	Insular Gyrus	174	1360	Dorsal caudate	228	627
	Globus Pallidus	222	1205	IPL	146	530
	IPL	146	1105	STG	76	517
	STG	80	1084	Insular Gyrus	170	322
	Insular Gyrus	170	820	Putamen	226	307
	Dorsal caudate	228	735	STG	78	81
	Postcentral Gyrus	158	716	STG	72	59
	MTG	82	696	pSTS	122	46
	STG	74	645	MTG	82	26
	STG	72	496	Postcentral Gyrus	158	12
	pSTS	122	443	pSTS	124	11
	IPL	136	292	Nucleus accumbens	224	11
	Putamen	226	289	STG	74	8
	IPL	138	261	Insular Gyrus	166	8
	MTG	86	229	Insular Gyrus	174	2
	Insular Gyrus	168	226	Ventral caudate	220	2
	ITG	100	211	MTG	84	1
	LOcC	210	133	MTG	86	1
	LOcC	202	117	IPL	142	1
	STG	76	100			
	IFG	38	74			
	Precentral Gyrus	62	70			
	IPL	140	63			
	IPL	144	60			
	Precentral Gyrus	54	47			
	STG	78	31			
	Postcentral Gyrus	160	25			
	Postcentral Gyrus	156	24			
	Nucleus accumbens	224	20			
	IPL	142	18			
	Insular Gyrus	166	16			
	pSTS	124	9			
	ITG	92	8			
	MVOcC	198	7			
	LOcC	200	6			
	Amygdala	214	5			
	Precuneus	152	3			
	SPL	130	2			
	Ventral caudate	220	1			
White matter (JHU)	External capsule	33	3066	External capsule	33	1245
	Superior corona radiata	25	1764	Internal capsule	17	1156
	SLF	41	1479	Posterior thalamic radiation	29	332
	Internal capsule	17	1110	SLF	41	325
	Posterior corona radiata	27	612	Internal capsule	21	285
	Internal capsule	21	501	Internal capsule	19	164
	Internal capsule	19	461	Superior corona radiata	25	161
	Anterior corona radiata	23	457	SFOF	43	147

SFOF	43	306	Posterior corona radiata	27	49
Posterior thalamic radiation	29	185	Uncinate fasciculus	45	25
Sagittal stratum	31	25	Sagittal stratum	31	4
Uncinate fasciculus	45	17			
Tapetum	47	10			

Note. Table lists cortical brain regions and white matter tracts that were covered by the five most important principal components (preselected via feature selection filter) predictive for chronic neglect severity (left column, components derived from whole-brain lesion maps) or direct improvement from acute to chronic stage of stroke (right column, components derived from lesion maps overlapping with the chronic neglect ROI). Gray matter regions and their labels are based on the Brainnetome Atlas (BNA; Fan et al., 2016) with 246 parcels; white matter tracts and their labels are based on the JHU ICBM white matter atlas (Mori et al., 2008) with 48 tracts. Regions are sorted by decreasing number of overlapping 1mm isotropic voxels (*N*).

References

Grasso, M. G., Troisi, E., Rizzi, F., Morelli, D., & Paolucci, S. (2005). Prognostic factors in multidisciplinary rehabilitation treatment in multiple sclerosis: An outcome study. Multiple Sclerosis Journal, 11(6), 719–724. https://doi.org/10.1191/1352458505ms1226oa

Fan, L., Li, H., Zhuo, J., Zhang, Y., Wang, J., Chen, L., Yang, Z., Chu, C., Xie, S., Laird, A. R., Fox, P. T., Eickhoff, S. B., Yu, C., & Jiang, T. (2016). The Human Brainnetome Atlas: A New Brain Atlas Based on Connectional Architecture. Cerebral Cortex, 26(8), 3508–3526. https://doi.org/10.1093/cercor/bhw157

Karnath, H.-O., Rennig, J., Johannsen, L., & Rorden, C. (2011). The anatomy underlying acute versus chronic spatial neglect: A longitudinal study. Brain: A Journal of Neurology, 134(Pt 3), 903–912. https://doi.org/10.1093/brain/awq355

Mori, S., Oishi, K., Jiang, H., Jiang, L., Li, X., Akhter, K., Hua, K., Faria, A. V., Mahmood, A., Woods, R., Toga, A. W., Pike, G. B., Neto, P. R., Evans, A., Zhang, J., Huang, H., Miller, M. I., van Zijl, P., & Mazziotta, J. (2008). Stereotaxic white matter atlas based on diffusion tensor imaging in an ICBM template. NeuroImage, 40(2), 570–582. https://doi.org/10.1016/j.neuroimage.2007.12.035

Shah, S., Vanclay, F., & Cooper, B. (1990). Efficiency, effectiveness, and duration of stroke rehabilitation. Stroke, 21(2), 241–246. https://doi.org/10.1161/01.STR.21.2.241
